# Implementation of the Surviving Sepsis Campaign in Patients With Heart Failure: Gender-Specific Outcomes

**DOI:** 10.7759/cureus.9140

**Published:** 2020-07-11

**Authors:** Baher Al Abbasi, Pedro Torres, Fergie Ramos-Tuarez, Kai Chen, Gustavo Avila, Endri Ceka, Andres R Chacon, Gretchen De Diego, Charles R Bornmann, Waqa Ghumman, Robert Chait, Jesus E Pino

**Affiliations:** 1 Internal Medicine, University of Miami/John F. Kennedy (JFK) Medical Center, Atlantis, USA; 2 Cardiovascular Medicine, University of Miami/John F. Kennedy (JFK) Medical Center, Atlantis, USA

**Keywords:** infection, fluids, mortality, cardiomyopathy, gender

## Abstract

Background

Limited data exist about the impact of gender-specific outcomes in patients with heart failure (HF) who develop concomitant sepsis.

Methods

This is a retrospective cohort study of patients with HF who developed sepsis. Clinical outcomes, including in-hospital mortality, development of cardiogenic shock (CS), pulmonary edema requiring urgent intravenous diuretics (IVD), acute kidney injury (AKI), length of stay (LOS), and 30-day HF-related readmission, were evaluated in men vs. women.

Results

This cohort of 618 patients includes 272 (44%) women with a mean age of 75±14 years. Coronary artery disease (p<0.0001), diabetes mellitus (p=0.0213), stage ≥ 3 chronic kidney disease (p<0.0001), and HF with reduced ejection fraction (HFrEF) (p=0.0015) were more prevalent in men. The implementation of the Surviving Sepsis Campaign (i.e., intravenous (IV) crystalloids in the first six hours) was more aggressive in women (p=0.0192). There was no difference in in-hospital mortality (p=0.2385) between men and women. After adjusting for HF types, women with HF with preserved ejection fraction (HFpEF) developed more episodes of pulmonary edema requiring urgent IVD (p=0.0389), while men with HFpEF had more CS requiring inotropes (p=0.0400) and a longer LOS (p=0.0434). Conversely, women with HFrEF were most likely to develop CS requiring inotropes (p=0.0132).

Conclusion

Women with HF who developed sepsis receive a more aggressive implementation of the Surviving Sepsis Campaign than men, leading to more pulmonary edema events in women with HFpEF and more cardiogenic shock in women with HFrEF. A cautiously tailored approach is desperately needed for patients with HF who develop sepsis.

## Introduction

Heart failure (HF) and sepsis frequently coexist, and they are associated with significant morbidity, mortality, and hospital cost. With a steady increase per year, more than 5.7 million people in the United States are affected by HF, and more than 1.5 million people are diagnosed with sepsis each year [1-5]. Importantly, the risk factors, pathophysiology, and quality of care of each condition differ significantly between genders [6]. For example, HF with preserved ejection fraction (HFpEF) is more common amongst elderly, obese women, and it is associated with higher morbidity, lower health-related quality of life, and more significant impairment in activities of daily living than amongst men [7-9]. Furthermore, despite representing more than half of all HF cases, women remain underrepresented in all HF trials. This leads us to inconclusive results that genuinely represent outcomes in this population [6,10-12].

Similarly, men and women have significant differences when affected by sepsis. Women have a higher ratio of anti-inflammatory to pro-inflammatory mediators, which has been hypothetically associated with a lower incidence of sepsis-related mortality than their counterparts [4,13].

To date, there is no current data regarding the impact of gender on clinically relevant outcomes and quality of life of men and women with HF who develop sepsis.

This study aims to evaluate the epidemiological characteristics, treatment choices (Surviving Sepsis Campaign), and outcomes of men and women with heart failure and sepsis.

## Materials and methods

We conducted a retrospective cohort study in a tertiary cardiovascular center between January 2015 and June 2018. Our patient population included those 18 years of age or older with a concomitant diagnosis of HF and sepsis. All patients were evaluated for baseline characteristics and relevant outcomes as an entire population. Subsequently, patients were stratified based on gender into men and women. For this study, heart failure was dichotomized into preserved ejection fraction (EF ≥50%) and reduced ejection fraction (EF <50%).

HF was defined based on signs and symptoms (i.e., congestion, edema, fatigue), laboratory data (i.e., elevated brain natriuretic peptide or NT-proBNP), and comprehensive echocardiographic evaluation that demonstrated systolic or diastolic dysfunction.

Sepsis was defined as a suspected infection and either ≥ two systemic inflammatory response syndrome (SIRS) criteria or ≥ two Quick Sepsis Related Organ Failure Assessment (qSOFA) criteria. SIRS include body temperature ≥ 38°C or ≤ 36°C, heart rate >90/min, respiratory rate ≥ 20/min or PaCO_2_ ≤ 32 mmHg, and white blood cell (WBC) count ≥ 12,000 cells/μL or ≤ 4,000/μL. qSOFA includes systolic blood pressure ≤100 mmHg, respiratory rate ≥ 22 breaths per min, or altered mentation with a Glasgow coma scale <15.

Data collection

An extensive chart review of clinically relevant variables, including baseline characteristics, blood cultures, urine cultures, sputum cultures, the use of antimicrobials, the type and rate of intravenous fluids, and the presence of underlying HF was performed.

Outcomes

The evaluated primary outcome is all-cause in-hospital mortality or discharge to hospice. Secondary outcomes included the development of pulmonary edema requiring urgent intravenous diuretics (IVD), development of cardiogenic shock (CS), post-admission acute kidney injury (AKI), hospital length of stay (LOS), and 30-day HF-related readmission rate.

Statistical analyses

Statistical analyses were performed using the JMP program version 14.0.0 (SAS Institute, Cary, North Carolina). Continuous variables are expressed as mean with standard deviation. A comparison between means was made using either two-sample T-tests, analysis of variance (ANOVA) (F statistic), or Pearson correlation. Results were considered significant if p-values <0.05. Regression models were used for primary outcomes to adjust for common cofounders. The institutional review board at JFK Medical Center and the University of Miami approved this study.

## Results

This cohort of 618 patients includes 272 (44%) women with a mean age of 75±14 years. A total of 439 (71%) patients have a history of chronic HF, and 179 (29%) are newly diagnosed with HF during the index hospitalization.

As demonstrated in Table 1, men were more likely to have a history of diabetes mellitus (p=0.0213), coronary artery disease (p<0.0001), coronary artery bypass graft surgery (CABG) (p<0.0001), HF with reduced ejection fraction (HFrEF) (p=0.0015), and stage >3 chronic kidney disease (CKD) (p<0.0001). Women were more likely to have higher body mass indices (p=0.0202) and HFpEF (p=0.0015). Biochemical abnormalities, such as leukocytosis, lactic acidosis, and elevated N-terminal (NT)-pro hormone BNP (NT-pro-BNP), were similar between genders (p=NS). Men were more likely to have elevated blood urea nitrogen (p=0.0005), creatinine (p=0.0002), and total bilirubin (p=0.0338). The respiratory system was the most common source of infection for both populations, but women had a higher proportion of genitourinary infections than men. Blood cultures demonstrated bacterial growth in 424/618 (68%) patients; however, there were no differences among genders (0.4856).

**Table 1:** Gender-specific differences in patients with heart failure and sepsis: baseline characteristics *Statistically significant; + plus-minus standard deviation; ¶ Heart failure with reduced ejection fraction (EF <50%); † Heart failure with preserved ejection fraction (EF >50%); § BNP: Brain natriuretic peptide; ‡ High-risk qSOFA (≥2 of 3 features that include respiratory rate >22, systolic blood pressure (SBP) <100, and altered mental status) suggests a high risk of poor outcomes in patients with suspected infection

N: 618	Women, N:272	Men, N:346	p-value
Characteristics			
Age, years ± SD^+^	76±14	74±14	0.1575
Race, Caucasians (%)	198(73)	275(79)	0.1532
Body mass index, kg/m^2 ^± SD^+^	28.6±7	27.2±5	0.0202*
Diabetes mellitus (%)	103(38)	163(47)	0.0213*
Hypertension (%)	231(85)	300(87)	0.5280
Coronary artery disease (%)	119(43)	211(61)	<0.0001*
Coronary artery bypass graft surgery (%)	20(7)	66(19)	<0.0001*
Chronic kidney disease stage >3 (%)	53(19)	119(34)	<0.0001*
Hemodialysis (%)	16(5)	35(10)	0.0576
Atrial fibrillation/flutter (%)	105(38)	146(42)	0.3665
Malignancy (%)	44(16)	48(13)	0.4245
High-risk qSOFA^‡^ (%)	120(45)	149(44)	0.7200
Heart failure types			
Ejection fraction, %	51.8±15	48±15	<0.0026*
Chronic heart failure (%)	198(72)	241(70)	0.3928
Reduced ejection fraction^¶^ (%)	84(31)	150(43)	0.0015*
Preserved ejection fraction^†^ (%)	188(69)	196(56)	
Vital signs			
Systolic blood pressure, mmHg ±SD^+^	110±35	111±30	0.8842
Temperature, Celsius ±SD^+^	37.2±0.9	37.2±0.9	0.4209
Heart rate, bpm ±SD^+^	99±24	97±23	0.3719
Respiratory rate ±SD^+^	20±4.7	20±4.7	0.9030
Laboratory data			
White blood counts x10^3^/μL ± SD^+^	15±8	14±8	0.3345
Hemoglobin, g/dL ± SD^+^	10±2	11±2	0.0891
Sodium, mEq/L ± SD^+^	138±6	137±6	0.5626
Blood urea nitrogen, mg/dL ± SD^+^	37±21	44±29	0.0005*
Creatinine, mg/dL ± SD^+^	1.6±0.9	2±1.2	0.0002*
Bilirubin, mg/dL ± SD^+^	0.9±1	1.3±2	0.0338*
Lactic acid >4 mmol/L (%)	65 (23)	66(19)	0.4038
Pro-BNP^§ ^X1000 ± SD^+^	17±25	20±40	0.3122
Troponin ng/mL ± SD^+^	0.5±1.5	0.5±1.5	0.8351
Albumin mg/dL ± SD^+^	2.5±0.7	2.4±0.6	0.7161

Predictors of outcomes such as high-risk qSOFA (p=0.7200), lactic acid >4 mmol/L(p=0.4038), and elevated ADHERE (Acute Decompensated Heart Failure National Registry) score (p=0.1023) were similar between men and women.

Management

All patients underwent comprehensive diagnostic testing, including biochemistry, cultures, and diagnostic imaging. Furthermore, the Surviving Sepsis Campaign was implemented in all of the patients (i.e., broad-spectrum antimicrobials in the first hour of the initial encounter and varying amounts of intravenous crystalloids). Women were more likely to receive aggressive intravenous crystalloids as a bolus and in the first six hours than men (22 ±14 ml/k vs. 19 ±14, p=0.0567 and 32 ±19 vs. 27±18 p=0.0192, respectively). After adjusting for the types of HF (HFrEF or HFpEF) and CKD (stage >3), women with HFpEF received a higher volume of intravenous crystalloids than men with HFpEF (22 ±14 ml/kg vs. 19 ±14, p=0.0397). Similarly, women without CKD received a higher amount of intravenous crystalloids fluid when compared to men 23±14 vs. 19±12 ml/kg (p=0.0013). Men with CKD received a more significant amount of intravenous crystalloids than women with CKD (23±14 vs. 19±12 p=0.0372). The amounts of intravenous crystalloid as a bolus and in the first six hours were not statistically different between men and women with HFrEF (p=0.6232, p=0.2734, respectively); see Table 2.

**Table 2:** Gender-specific differences in patients with heart failure and sepsis: intravenous crystalloid fluid management + plus-minus standard deviation; *Outcomes were statistically significant; ‡Reduced ejection fraction (EF) defined as EF <50%; †Preserved ejection fraction defined as EF >50%

N=618	Women, N: 272			Men, N: 346		p-value
Bolus, ml/kg ± SD^+^	22±14			19±14		0.0567
6 hours, ml/kg ± SD^+^	32±19			27±18		0.0192*
	Preserved EF^†^			Reduced EF^‡^		
	Women	Men	p-value	Women	Men	p-value
Bolus, ml/kg ± SD^+^	22±14	19±14	0.0397*	21±14	19±14	0.6232
6 hours, ml/kg ± SD^+^	32±19	27±18	0.0262*	31±21	28±20	0.2734
	CKD stage >3			No CKD stage >3		
	Women	Men	p-value	Women	Men	p-value
Bolus, ml/kg ± SD^+^	13±1.8	21±1.5	0.0372*	23±14	19±12	0.0013*

Outcomes

In-hospital mortality or discharge to hospice occurred in 193 (31%) patients. There was no difference in the mortality between men and women with HF who developed sepsis (p=0.2385); see Table 3.

**Table 3:** Heart failure and sepsis: gender-specific outcomes + plus-minus standard deviation; IV: Intravenous; AKI: Acute kidney injury

N=618	Women; N:272	Men.; N:346	p-value
In-hospital mortality or discharge to hospice care (%)	78(29)	115(33)	0.2385
Pulmonary edema requiring urgent IV diuretics (%)	176(65)	199(57)	0.0692
Post-admission AKI (%)	134(50)	177(52)	0.7285
Cardiogenic shock (%)	21(8)	25(7)	0.7936
Length of stay, days ± SD^+^	10±9	10±12	0.5434
30-day readmission (%)	40(22)	45(21)	0.8758

After adjusting for the types of HF (HFrEF or HFpEF), there was no significant difference in the primary outcome in this cohort (see Table 4).

**Table 4:** Gender-specific outcomes by heart failure types + plus-minus standard deviation; *Outcomes were statistically significant; ‡Reduced ejection fraction defined as EF <50%; †Preserved ejection fraction (EF) defined as EF >50%; IV: Intravenous; AKI: Acute kidney injury

N=618	Preserved ejection fraction^† ^N: 384			Reduced ejection fraction^‡. ^N: 234		
	Women N:188	Men N:196	p-value	Women N:84	Men N:150	p-value
In-hospital mortality or discharge to hospice care (%)	53(28)	55(28)	0.9500	25(30)	60(40)	0.1183
Pulmonary edema requiring urgent IV diuretics (%)	123(65)	108(55)	0.0389*	53(63)	91(60)	0.7141
Post-admission AKI (%)	91(50)	101(52)	0.6126	43(52)	76(51)	0.9069
Cardiogenic shock (%)	2(1)	9(5)	0.0400*	19(23)	16(11)	0.0132*
Length of stay, days ± SD^+^	9±8	11±11	0.0434*	12±11	9±13	0.1633
30-day readmission (%)	23(19)	27(22)	0.6125	17(29)	18(22)	0.3025

Secondary outcomes

Women with HFpEF developed more episodes of pulmonary edema, requiring urgent intravenous diuretics (p=0.0389), while men with HFpEF had more episodes of cardiogenic shock requiring inotropes (p=0.0400) and a longer LOS (p=0.0434). Conversely, women with HFrEF were most likely to develop cardiogenic shock (p=0.0132).

Unadjusted data showed no difference between men and women in the incidence of cardiogenic shock (p=0.7936), development of pulmonary edema requiring urgent IV diuretics (p=0.0692), post-admission AKI (p=0.7285), length of stay (p=0.5434), and 30-day HF-related readmission (p=0.8758).

## Discussion

Our study demonstrates that HF and sepsis are a dangerous combination. In this cohort, patients with HF and concomitant sepsis had a 31% mortality, which is six to 10 times higher than the mortality for each individual syndrome. This high mortality is explained by several pathophysiologic changes that occur in the cardiovascular system once a patient develops sepsis.

First, there is a surge of pro-inflammatory cytokines, leading to a decrease in systemic vascular resistance (SVR), a decrease in afterload, and an increase in cardiac output (CO). Additionally, there is an increase in sympathetic tone leading to fast heart rates (HR) and myocardial contractility that translates into higher oxygen consumption of the left ventricle (LV-max-Vo2) and a further increase in CO [14].

Importantly, in a patient with heart failure, the SVR, HR, and LV-max-Vo2 are chronically impaired [15]. Thus, when these patients with a diminished cardiovascular reserve face an acute event, such as sepsis, they are unable to meet the metabolic demands of the severely infected host, leading to catastrophic consequences and mortality.

Traditionally, it has been reported that women have lower sepsis-related mortality than men [4]. However, in this cohort, there was no difference in mortality between men and women with HF who develop sepsis. Women have a proportionally higher ratio of anti-inflammatory-to-pro-inflammatory cytokines, while men have higher levels of interleukin-6 [16]. This translates to a better, more coordinated, and more organized immune response to infection in women as well as lower mortality than in men. Furthermore, in our study, men had a higher prevalence of chronic comorbid conditions, such as coronary artery disease, CABG, CKD, HFrEF, and diabetes mellitus, which are known to be independent predictors for poor outcome and mortality for patients with sepsis regardless of the presence of heart failure or not. Therefore, despite having a better immunological response to infection, lower prevalence of HFrEF, and lesser comorbidities than men, women with heart failure that developed sepsis have similar mortality rates than men. This suggests that the presence of HF (a chronic debilitating condition) and its chronic neuroendocrine disarrangement are more critical on women than men.

Management

The Surviving Sepsis Campaign was implemented in all men and women in this population. Both groups received broad-spectrum antimicrobials and a similar rate of invasive and non-invasive mechanical ventilation. However, women received a higher volume of intravenous crystalloids than men, which subsequently translated to higher complication rates, such as pulmonary edema requiring urgent intravenous diuretics (women with HFpEF) and higher incidence of cardiogenic shock, requiring inotropes (women with HFrEF).

This difference in fluid administration between men and women could be explained in two ways; men had a higher prevalence of CKD and HFrEF, which led to more cautious fluid administration by providers. Additionally, there is a false belief that patients with HFpEF (more common in women) can tolerate a similar amount of fluid than the general population or more fluids than patients with HFrEF (more common in men). Regardless of the differences among sex groups, this study suggests that men and women with HF who develop sepsis have very different physiology, risk factors, and treatment approaches but with an ultimately similar risk of mortality. This highlights the need for a more physiologic approach of sepsis in patients with heart failure rather than standardized, protocol-driven interventions.

## Conclusions

Management of sepsis in patients with HF represents a challenging and complex clinical conundrum. In contrast to previously reported literature, women with HF who develop sepsis have similar mortality to men despite fewer comorbidities and fewer predictors of poor outcomes. Furthermore, women with HF who develop sepsis receive a more aggressive implementation of the Surviving Sepsis Campaign than men, leading to more volume overload-related complications. A cautiously tailored approach is desperately needed for this population.
